# STGFormer: Spatio-Temporal Graph Transformer for Traffic Flow Prediction in Sparse-Sensing Scenarios

**DOI:** 10.3390/s26165082

**Published:** 2026-08-11

**Authors:** Jin Zhang, Fengmin Tan, Wei Bai, Feiyang Peng, Weijie Wang

**Affiliations:** 1 National Innovation Center of Intelligent and Connected Vehicles, Beijing 100036, China; hitzhangjin@163.com (J.Z.); tangfengmin@china-icv.cn (F.T.); 2 Department of Road Traffic Management, Sichuan Police College, Luzhou 646000, China; 3 Intelligent Policing Key Laboratory of Sichuan Province, Luzhou 646000, China; 4 School of Transportation, Southeast University, Nanjing 211189, China; 220253404@seu.edu.cn (F.P.); 230259410@seu.edu.cn (W.W.)

**Keywords:** traffic flow prediction, transformer, expressway, graph convolutional network, weather factors

## Abstract

**Highlights:**

**What are the main findings?**

- An enhanced spatio-temporal graph Transformer (STGFormer) is proposed for traffic flow prediction, combining temporal self-attention and learnable temporal encoding to capture both long-term traffic evolution patterns and sudden fluctuations.
- Weather data are incorporated into the prediction process to achieve robust prediction under complex environmental conditions.

**What are the implications of the main findings?**

- An effective solution is provided for traffic flow prediction in sparse-sensing scenarios.
- The applicability of traffic prediction models is extended to complex and heterogeneous real-world environments.

**Abstract:**

With the continuous improvement in intelligent transportation and data perception levels, determining how to achieve high-precision and generalizable traffic flow prediction based on historical traffic data has become an important issue in intelligent highway management. The Transformer model, with its strong temporal modeling capabilities, has gradually become a research hotspot in time series prediction. However, its original structure has certain limitations in modeling spatial dependencies, making it difficult to fully exploit the topological relationships of the traffic network, and it has weak adaptability to external environmental changes. To address these issues, this paper proposes an improved Transformer prediction model that integrates a graph convolutional network (GCN) and a self-attention mechanism. The model captures spatial topological information through the GCN module, introduces temporal self-attention mechanism and temporal encoding to enhance the modeling of temporal features, and combines weather factors to achieve perception modeling of external disturbances. In the experimental design, considering the uneven distribution of perception resources in reality, the model input only uses the historical traffic data of some nodes, and different node coverage rates are set to test the performance of the model under sparse input conditions. The results show that the model can maintain good accuracy and stability under multiple coverage rates, verifying the effectiveness and application prospects of the structural improvement.

## 1. Introduction

With the rapid development of the economy, China’s transportation network has undergone continuous refinement. As a critical infrastructure supporting regional economic growth and urban agglomeration connectivity, the highway system has become increasingly complex in its network structure, placing higher demands on operational efficiency and management effectiveness [1]. As traffic volumes rise and the road network expands, the traditional “comprehensive sensing–centralized forecasting” paradigm has revealed limitations, including high sensing costs and limited predictive accuracy. Optimizing the allocation of sensing resources while ensuring forecasting precision has, thus, emerged as a central challenge in the development of intelligent transportation systems (ITSs).

Highway systems, as typical examples of heterogeneous traffic networks, exhibit operational dynamics that heavily rely on a small number of pivotal nodes within the network structure. Studies have shown that certain nodes play central roles in both the topological configuration and the spatio-temporal evolution of traffic flows. Constructing a monitoring sub-network based on these critical nodes has the potential to reduce sensing and computational costs while preserving the overall trend of traffic evolution. Kong et al. [2] proposed a scoring mechanism to identify key nodes in transportation networks and developed a Spatio-Temporal Pivotal Graph Neural Network (STPGNN) to capture the spatio-temporal dependencies between pivotal and nonpivotal nodes. They also designed a parallel recognition framework for both node types to enhance traffic flow prediction. Similarly, Li et al. [3] identified critical nodes through a minimum connected dominating set approach and formed a backbone sub-network composed of key nodes and significant edges to analyze air traffic flows. This approach introduced the concept of “key node coverage ratio” as an input control variable in prediction models, enabling generalization under sparse input conditions.

At the same time, traffic flow prediction—one of the core technologies underpinning ITS—has evolved from traditional statistical methods to deep-learning-based models. Conventional methods typically focus on time series characteristics or single-point data modeling, often overlooking the influence of pivotal nodes within the network topology. Consequently, these traditional approaches are gradually being replaced by machine learning techniques with stronger feature extraction and modeling capabilities, such as support vector regression (SVR), random forests, and neural networks.

### 1.1. Current Research Status

Traffic data exhibit multiple intricacies that challenge prediction models: (i) spatio-temporal coupling, where cross-sections that are topologically close exhibit strong correlation while distant or opposing-direction sections do not; (ii) non-Euclidean structure, as road networks form graphs rather than regular grids, requiring specialized convolution operators; and (iii) dual temporal patterns, combining long-term periodicity (e.g., daily and weekly cycles) with abrupt fluctuations triggered by incidents, weather changes, or demand surges.

In recent years, deep learning has achieved significant progress in traffic flow prediction. Recurrent neural networks (RNNs) have demonstrated superior performance in modeling time series, while convolutional neural networks (CNNs) effectively extract spatial structural information through local receptive fields. Li et al. [4], considering the spatio-temporal correlations and evolutionary features of traffic flow data, proposed a hybrid model combining CNNs and bidirectional long short-term memory networks (BiLSTMs). By transforming input data into three-dimensional matrices, the model utilizes CNNs to extract spatial features and BiLSTMs for temporal features, ultimately producing prediction results. Specifically, the CNN employs local receptive fields and shared convolution kernels to extract spatial correlations from the three-dimensional traffic-flow input, whereas the BiLSTM processes the CNN-generated feature sequence in both forward and backward directions to capture temporal dependencies within the historical observations. The resulting bidirectional temporal representation is then mapped to the traffic-flow prediction output. Yang et al. [5] enhanced the performance and generalizability of CNNs in traffic flow prediction by integrating multiple temporal and contextual features, such as periodic trends and external conditions, into a unified multifeature fusion framework. Zhang et al. [6] proposed an attention-based spatial–temporal model that combines CNNs for spatial encoding and GRUs for temporal decoding, effectively enhancing prediction performance across different input dimensions and time steps. Narmadha and Vijayakumar [7] developed a hybrid CNN–LSTM model, where CNNs extract upstream and downstream spatial features, and LSTMs capture temporal dependencies.

Given CNNs’ limitations in modeling the non-Euclidean spatial correlations inherent in road networks, graph convolutional networks (GCNs) have been introduced to enhance spatial modeling capabilities [8]. Zhao et al. [9] proposed a temporal graph convolutional network combining GCN and GRU modules to, respectively, capture spatial and temporal dependencies. Yin Ye et al. [10] introduced a dynamic spatio-temporal hypergraph convolutional network (DSTHGCN) that performs spectral hypergraph convolution and graph convolution jointly, incorporates a hyperedge outlier removal mechanism to refine the structure, and significantly improves traffic flow forecasting accuracy. Zheng et al. [11] introduced a graph-based multiattention network to forecast traffic conditions at various time steps, using dynamic spatio-temporal attention layers and a cross-domain mapping mechanism from encoded features to decoded sequences for effective temporal feature propagation. Additionally, some researchers have integrated gating mechanisms into GCNs [12,13], enabling joint spatio-temporal modeling to better capture the intricacies of traffic data.

Although CNNs and GCNs are effective in capturing local spatial features and topological dependencies, respectively, they still struggle with modeling complex spatio-temporal dynamics and long-term dependencies. The Transformer architecture, through its multihead self-attention mechanism and parallelized encoding structure, offers a novel approach for decoupling spatio-temporal dependencies. Its nonrecursive computation overcomes inherent limitations in traditional RNN/CNN-based models related to long-range dependency modeling and gradient propagation, making it a scalable framework for spatio-temporal sequence modeling tasks such as traffic forecasting and road network state inference. In recent years, various studies have explored the application of deep neural networks, including Transformer-based architectures, to short-term traffic flow prediction with promising results [14].

In recent years, Transformer-based models have gained substantial traction in traffic flow prediction due to their ability to model complex spatio-temporal dependencies. Moon and Cho et al. [15] introduced a graph Transformer model embedded with subgraph structures, which effectively captures local spatial dependencies by incorporating subgraph-based structural priors into the attention mechanism, leading to improved prediction accuracy across urban traffic datasets. Lin et al. [16] proposed LVSTformer, a layered-view spatio-temporal Transformer that enhances feature interactions across multiple levels of temporal and spatial representations, demonstrating strong performance in short-term forecasting scenarios. To address challenges in capturing both fine-grained and long-range dependencies, Tian et al. [17] developed MSSTAT, a multiscale spatio-temporal-aware Transformer framework that integrates hierarchical attention to learn from both short-term fluctuations and long-term traffic trends. Zhong et al. [18] presented MSPSTT, a multiscale persistent spatio-temporal Transformer, which focuses on long-term traffic flow prediction by modeling temporal persistence and dynamic city-scale variations. Li et al. [19] introduced DDGformer, a direction- and distance-aware graph Transformer that encodes directional traffic flow patterns and spatial distances directly into the attention mechanism, significantly enhancing the model’s ability to detect spatial heterogeneity in complex traffic networks. Xue et al. [20] developed an adaptive spatio-temporal Transformer network that utilizes a multiview temporal attention module to learn both short- and long-term temporal dependencies. By dynamically capturing spatial dependencies through self-attention, the model addresses issues caused by forecast time-step propagation. To synthesize the above literature and clarify the scope of the present study, Table 1 compares representative models in terms of their core modeling features and their treatment of sparse-sensing conditions and external factors.

As summarized in Table 1, existing studies have made substantial progress in spatial dependency modeling, temporal pattern extraction, and external-feature integration. CNN–RNN hybrid models can capture local spatial and temporal patterns but do not explicitly represent the non-Euclidean topology of road networks. Graph-based models provide stronger spatial representations, yet many assume complete observations over a predefined network and pay limited attention to prediction under sparse key-node coverage. Moreover, weather information, graph-based spatiotemporal dependencies, and incomplete node observations have rarely been examined within a unified prediction framework. Therefore, an integrated approach capable of jointly modeling road-network topology, long- and short-term temporal patterns, environmental disturbances, and sparse observations is still needed.

### 1.2. Research Objectives and Contributions

To address these research gaps, this study proposes STGFormer, a Spatio-Temporal Graph Transformer designed for traffic-flow prediction under sparse-sensing scenarios. The main contributions of this study are summarized as follows:

1. Unified spatio-temporal dependency modeling. A two-layer GCN is integrated with a temporal self-attention mechanism and temporal encoding. The GCN captures direct and higher-order spatial dependencies among gantry nodes, while the temporal modules model both long-range periodic patterns and abrupt traffic-flow fluctuations.
2. Prediction under sparse-sensing conditions. The proposed model uses historical observa-tions from selected key nodes to infer traffic flow across the complete study corridor. Mul-tiple key-node coverage rates are designed to evaluate prediction accuracy, stability, and generalization under different sensing-resource constraints.
3. Weather-aware feature fusion. Weather conditions and rainfall are incorporated as exter-nal disturbance variables and fused with the spatial features extracted by the GCN, im-proving the representation of traffic-flow variations under changing environmental condi-tions and limited node observations.

## 2. Traffic Flow Prediction Model Based on Enhanced Transformer

In the development of traffic state inference techniques, early studies were often constrained by decoupled modeling frameworks, treating spatial and temporal dimensions in isolation. In response to this limitation, this study proposes the spatio-temporal graph Transformer (STGFormer) model framework. STGFormer integrates graph convolutional networks (GCNs) to extract spatial features and model spatial dependencies, while leveraging the Transformer’s strength in capturing temporal correlations through a dedicated time encoding module. A self-attention mechanism is employed to learn the temporal evolution of traffic flows across multiple time scales, effectively modeling time dependencies. Furthermore, weather data are integrated with spatial features, overcoming the limitations of static feature fusion in traditional approaches. The input to STGFormer consists of data from key nodes selected via a node-importance identification method. Through deep spatio-temporal feature fusion, the model enables traffic flow prediction for all nodes across the entire road network.

To improve the readability of the mathematical notation used in the proposed STGFormer framework, the principal symbols and indices are summarized in Table 2.

### 2.1. Model Architecture Overview

**Figure 1:**
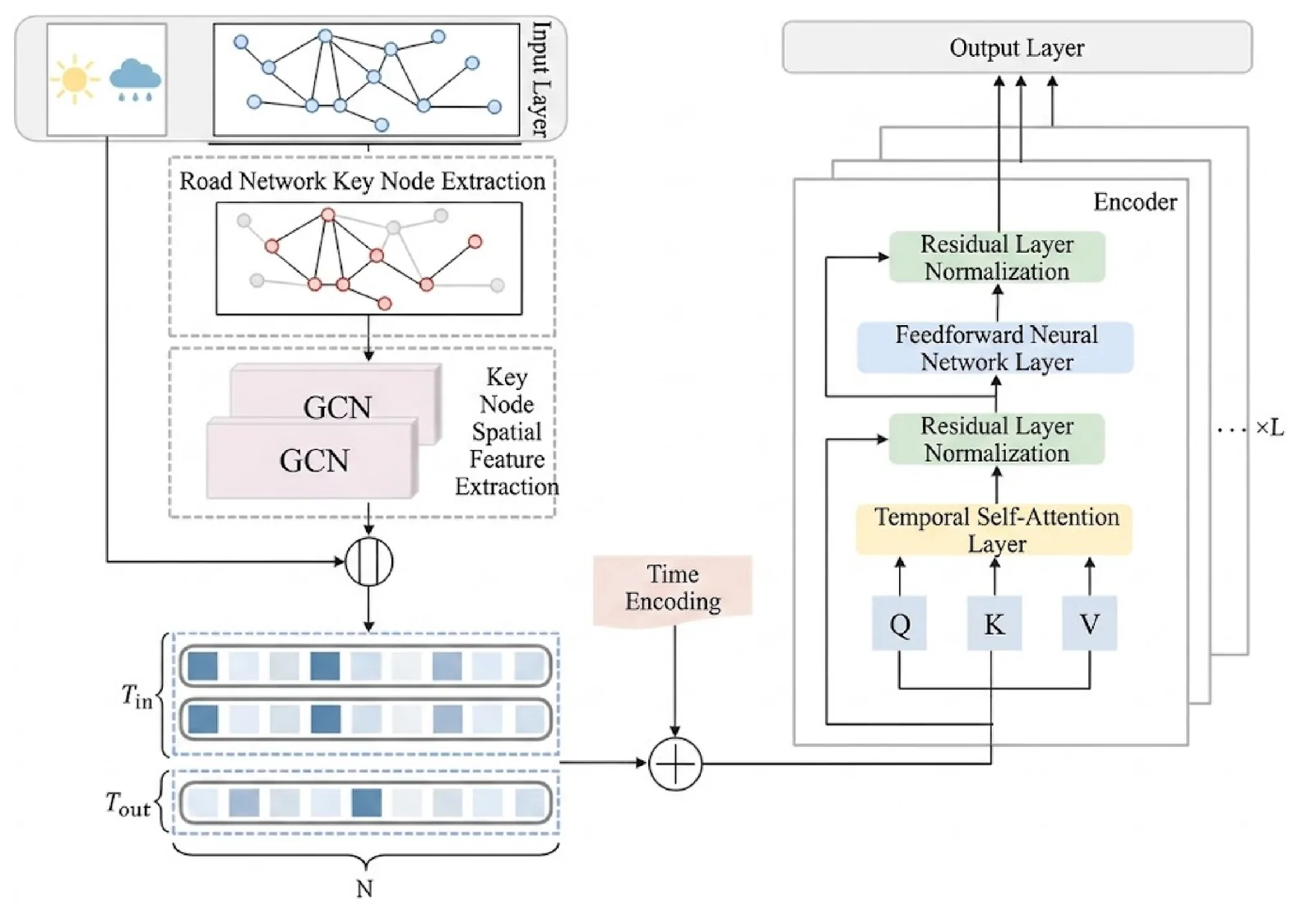
Architecture of the STGFormer model.

The proposed model adopts a dual-layer cascaded GCN structure to analyze the spatial features of input data from key nodes. Since the inputs to the GCN are limited to a subset of critical nodes, the model dynamically adjusts to varying key node coverage ratios. As the coverage ratio changes, the spatial convolution weights and information propagation paths adapt accordingly, allowing the GCN to flexibly capture the spatial topology of the input. At the output layer of the spatial feature extraction module, weather data are fused with the spatial features and passed as input to the temporal feature extraction module. In the temporal modeling process, a self-attention mechanism based on a sliding time window is employed to construct input–target sequence pairs for the prediction of future time steps. Unlike traditional recursive prediction methods, this approach supports parallel multistep forecasting, improving both accuracy and computational efficiency. The overall architecture of the proposed STGFormer model is illustrated in Figure 1. Specifically, the input traffic flow data are organized as a three-dimensional tensor $X\in R^{T_{in}\times N_{k}\times F}$, where $T_{in}$, $N_{k}$, and F denote the number of historical time steps, selected key nodes, and input features, respectively. The input tensor is mapped by a fully connected layer into the latent representation $H_{gcn,t}^{\left(0\right)}$, which is subsequently processed by the two-layer GCN to obtain the spatial feature matrix $H_{gcn,t}^{\left(2\right)}$. The weather feature matrix $S^{W}$ is then fused with the spatial features and fed into the temporal self-attention module. Finally, the model generates the prediction tensor $\hat{Y}\in R^{T_{out}\times N\times 1}$, where $T_{out}$ and N represent the number of prediction time steps and all nodes in the road network, respectively.

Firstly, the input traffic flow data is passed through a fully connected layer for a linear transformation, mapping the single feature of each node into a high-dimensional latent space, as shown in Equation (1).

(1) $$h_{i}^{\left(0\right)}=Wx_{i}+b$$

In the equation, $x_{i}$ denotes the traffic flow feature of a node; W represents the learnable weight parameters; b is the bias term. The output is a node feature representation with a dimensionality of $d_{gcn}$.

In the spatial feature extraction module, the node features at each time step are passed through two layers of GCN to capture spatial dependencies. The propagation rule of the GCN is defined as shown in Equation (2).

(2) $$h_{i}^{\left(l+1\right)}=\sigma \left(\sum_{j\in N(i)\cup \{i\}}\;\frac{1}{\sqrt{d_{i}d_{j}}}h_{j}^{\left(l\right)}W^{\left(l\right)}+b^{\left(l\right)}\right)$$

In Equation (2), N(i) denotes the set of neighboring nodes of node i; $d_{i}$ denotes the degree of node i; $W^{\left(l\right)}$ and $b^{\left(l\right)}$ are the learnable parameters at the l-th GCN layer; and σ(⋅) denotes the activation function, for which ReLU is adopted.

The output is the node feature matrix at each time step.

Weather features are encoded using one-hot encoding to vectorize discrete weather parameters. Based on a time-slicing mechanism, the resulting weather feature matrix at each time step is denoted as $S_{t}^{W}$, where each time slice’s weather attributes are mapped using orthogonal basis vectors. In the feature fusion module, the weather features are first dimensionally expanded and then concatenated with the node features extracted from the spatial feature extraction module along the feature dimension, as expressed in Equation (3).

(3) $$z_{t}=concat\left(h_{t},x_{t}^{weather}\right)$$

In the equation, $h_{t}$ denotes the extracted node features; $x_{t}^{weather}$ represents the weather features.

The concatenated feature vector is then passed through a fully connected layer for a linear transformation, adapting its high-dimensional latent representation to match the input dimension required by the Transformer encoder module.

Based on the temporal characteristics of both historical observations and the prediction horizon, the system pregenerates time encoding vectors, which are subsequently fused with the latent traffic flow features through an element-wise addition operation, as shown in Equation (4).

(4) $$H_{enc}^{\left(0\right)}=\left[\left(WX+b\right)∥Z\right]+TEC$$

In the equation, $H_{enc}^{\left(0\right)}\in R^{T\times N_{k}\times h_{d}}$, $N_{k}$ is the number of selected key nodes, $T=T_{in}+T_{out}$ is the length of the time window, $T_{in}$ denotes the historical sequence length, $T_{out}$ is the prediction horizon, $h_{d}$ is the dimension of hidden features; W and b are learnable parameters; Z represents a zero-padding matrix used to align the input dimension for the encoder; ∥ denotes the concatenation operation; TEC is the time encoding vector.

In the temporal feature extraction module, the encoder takes the hidden representations of the traffic flow sequence $H_{enc}^{\left(0\right)}$ as its initial input. The encoder adopts an $L_{e}$-layer stacked architecture, where each layer shares a similar structure but has independently learned parameters. Each layer consists of a temporal attention mechanism and a feedforward neural network (FFN). The temporal attention layer is responsible for integrating temporal dependencies, and its parameters are shared across all nodes at each time step. The FFN constructs nonlinear mappings through a multilayer perceptron, effectively enhancing the model’s expressive capacity. Furthermore, a temporal masking matrix $M_{mask}$ is incorporated into the temporal attention mechanism to ensure that the attention is restricted to the current and past time steps, thereby preventing the model from accessing future information during prediction. The formulation of the temporal masking matrix is provided in Equation (5).

(5) $$M_{mask}\left[i,j\right]=\left\{\begin{array}{c} 0,\;\;\;\;\;\;\;\;\;\;\;\;\;\;j\leq i\\ -\infty ,\;\;\;\;\;\;\;\;\;i<j \end{array}\right.$$

The temporal feature extraction module comprises this $L_{e}$-layer Transformer encoder, where each layer contains a temporal self-attention sub-layer (with parameters shared across all nodes at each time step) and a position-wise feedforward network. The temporal masking matrix in Equation (5) ensures causality by restricting attention to current and past time steps, preventing information leakage from future observations during training. This architecture enables the model to capture both long-range dependencies and abrupt fluctuations in traffic flow. The model inherits the architectural design of the Transformer, maintaining residual connections and layer normalization across its components. The encoder layers are stacked sequentially, employing the same connection mechanisms between layers. The final output of the model is passed through a fully connected layer that maps the feature dimensions back to the dimension corresponding to the actual number of nodes, producing predictions for the predefined forecasting horizon.

### 2.2. Spatial Dependency Modeling Based on Graph Convolutional Networks

GCNs offer a way to capture spatial correlations among nodes. They have benefits like high computational efficiency, fewer parameters, and good performance even with limited samples. Yet, they depend on a fixed road topology graph. Given this study’s focus on predicting traffic flow across entire routes using some key nodes, the chosen nodes might make the road network sparse. Therefore, it is essential to capture higher-order neighborhood information. This can extract richer spatial features and better handle sparse graph structures. Ultimately, to balance performance and computational complexity, this study uses a two-layer GCN for spatial dependency modeling.

The process starts by building a traffic graph topology from key nodes. Generally, the smaller the distance between road nodes, the closer their connection. Conversely, when the distance exceeds a certain threshold, their mutual influence becomes negligible. This paper constructs the road topology graph based on node spacing. It uses distance-based Gaussian kernel edge weights (Equation (6)). Unlike simple inverse distance, this method gives a smoother and more reasonable pattern of spatial influence decay.

(6) $$\omega_{ij}=\exp\left(-\frac{{distance\left(v_{i},v_{j}\right)}^{2}}{2{\sigma_{g}}^{2}}\right)$$

In Equation (6), $distance\left(v_{i},v_{j}\right)$ is the Euclidean distance between nodes i and nodes j; $\sigma_{g}$ denotes the bandwidth parameter of the Gaussian kernel. The next step is to build a spatial feature extraction module, which deeply aggregates spatial features between key nodes by constructing a renormalized graph propagation matrix. This mathematical representation establishes an efficient integration mechanism for road network node information in the spatial dimension. Specifically, the traffic flow data processed by graph convolution at time *t* can be represented by Equation (7).

(7) $$X_{t}^{\prime }=\tilde{L}X_{t}W$$

In Equation (7), $\tilde{L}$ is the renormalized graph propagation matrix; $X_{t}$ is the traffic flow data at time t; W is the learnable parameter.

**Figure 2:**
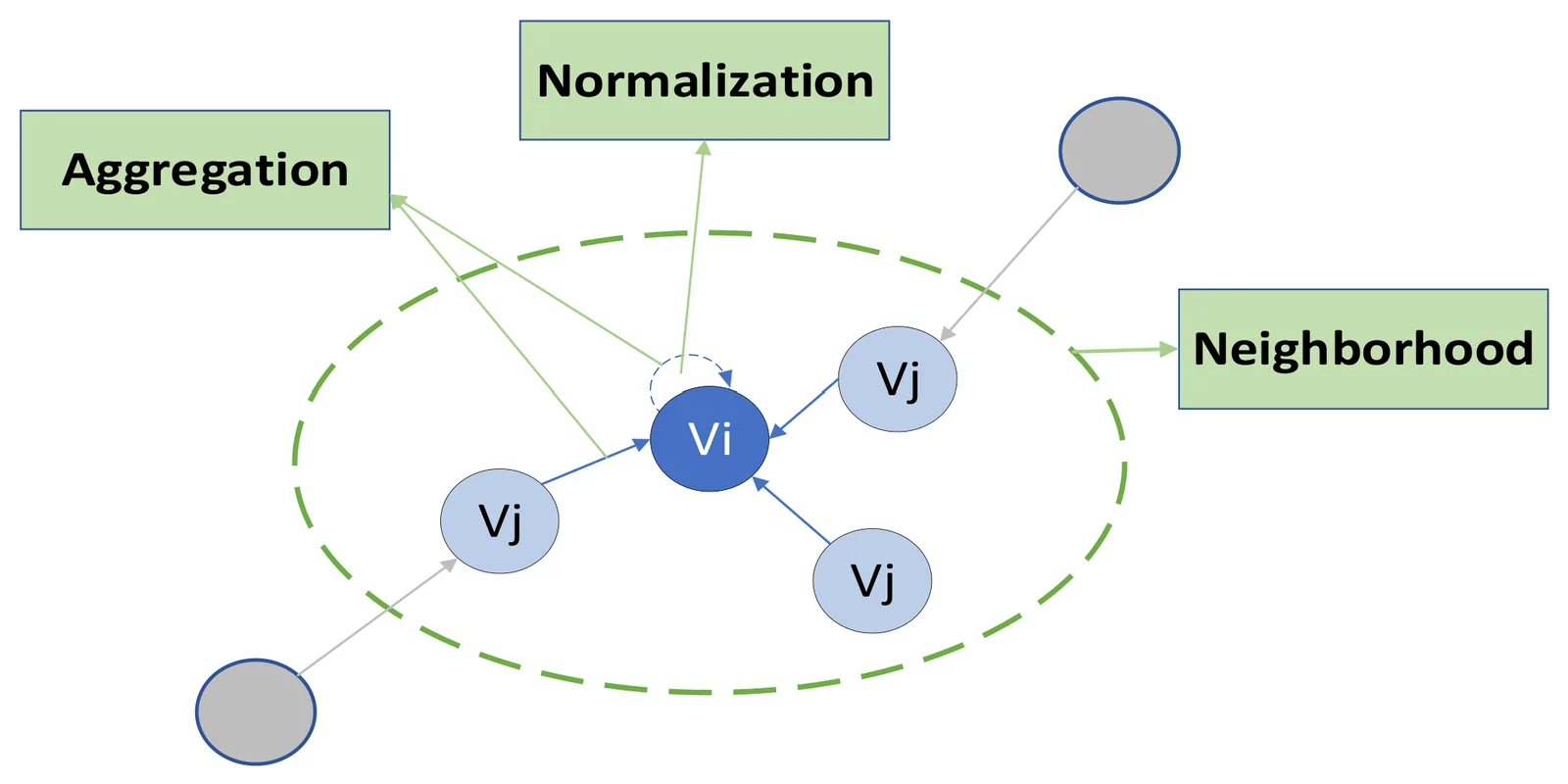
Graph convolutional network for extracting spatial features.

The entire feature extraction process is illustrated in Figure 2. When extracting node features, the GCN essentially calculates the weighted sum of local neighborhood features around a central point. For the central node, its neighborhood is defined as a topological collection that includes itself and its directly connected neighbors. The aggregation weights for each node are determined by the corresponding edge weights.

Notably, differences in node degree distribution can lead to feature scale shifts. For instance, high-degree nodes would cause feature values to abnormally expand or explode, while low-degree nodes would produce vanishing features, causing the model training process to diverge. To address this, the renormalized graph propagation matrix ${\tilde{D}}_{c}^{-1/2}\tilde{A_{c}}{\tilde{D}}_{c}^{-1/2}$ is employed here to ensure stable model training through symmetric normalization, which keeps each node’s aggregated representation numerically stable regardless of its connectivity—a property that is critical in sparse graph structures where degree variance is high. Furthermore, the GCN can be used either independently or stacked across multiple layers to capture higher-order neighborhood information. The layer-wise propagation rule is shown in Equation (8).

(8) $$H^{\left(l+1\right)}=\sigma \left({\tilde{D}}_{c}^{-1/2}\tilde{A_{c}}{\tilde{D}}_{c}^{-1/2}H^{\left(l\right)}W^{\left(l\right)}\right)$$

In Equation (8), $A_{c}$ denotes the weighted adjacency matrix of the key-node graph; ${\tilde{A}}_{c}=A_{c}+I_{N_{k}}$ is the self-connected adjacency matrix; ${\tilde{D}}_{c}$ is the degree matrix of ${\tilde{A}}_{c}$, satisfying ${\tilde{D}}_{c,ii}=\sum_{j}{\tilde{A}}_{c,ij}$; $W^{\left(l\right)}$ is the trainable weight matrix at the l-th layer; σ(⋅) denotes the nonlinear activation function; and l is the layer index.

This study adopts a two-layer GCN to capture multihop spatial dependencies. The first layer captures direct first-order correlations between adjacent gantries, while the second layer further aggregates indirect second-order influences. Such a design enables the model to effectively infer global traffic patterns even under sparse key node input, which is critical for real-world sparse sensing scenarios. As shown in Algorithm 1, spatial feature extraction is followed by weather-feature fusion.
**Algorithm 1.** Spatial feature extraction and weather-feature fusionFor a given key node coverage rate c, let $V_{c}$ denote the selected key node set, and let $N_{k}=|V_{c}|$.**Input:** Historical traffic-flow tensor $X\in R^{T_{in}\times N_{k}\times F}$; selected key node set $V_{c}$, weather-feature sequence $S^{W}$, and Gaussian-kernel parameter $\sigma_{g}$.**Output:** Fused feature sequence $\{z_{t}{\}}_{t=1}^{T_{in}}$.Construct the road-network topology among the selected key nodes according to their spatial positions and traffic directions.Calculate the spatial weight $\omega_{i,j}$ between nodes $v_{i}$ and $v_{j}$ using the distance-based Gaussian kernel in Equation (6).Construct the weighted adjacency matrix $A_{c}$, add self-connections as $\tilde{A_{c}}=A_{c}+I_{N_{k}}$, and calculate the corresponding degree matrix ${\tilde{D}}_{c,ii}=\sum_{j}\;{\tilde{A}}_{c,ij}$.Construct the renormalized graph propagation matrix $\tilde{L}={\tilde{D}}_{c}^{-1/2}\tilde{A_{c}}{\tilde{D}}_{c}^{-1/2}.$**For** each historical time step $t=1,\ldots ,T_{in}$ **do**
Obtain the initial node representation $H_{gcn,t}^{\left(0\right)}$.**For** each GCN layer l=0,1 **do**
Aggregate the features of each node and its neighbors according to Equations (2), (7), and (8).Apply the ReLU activation function to obtain $H_{gcn,t}^{\left(l+1\right)}$.
**End for**Take $H_{t}=H_{gcn,t}^{\left(2\right)}$ as the spatial representation at time step t.Expand the weather feature matrix $S_{t}^{W}$ along the node dimension to obtain $x_{t}^{weather}$.Fuse the spatial and weather features according to Equation (3)
**End for**Return the fused feature sequence $\{z_{t}{\}}_{t=1}^{T_{in}}.$

### 2.3. Temporal Dependency Modeling Based on Attention Mechanisms

Temporal dependency modeling with attention mechanisms hinges on two keys: building a time self-attention mechanism and designing time encoding.

#### 2.3.1. Constructing the Time Self-Attention Mechanism

This study selects and designs a dynamic temporal self-attention architecture to analyze the cross-period dependency characteristics in traffic state evolution. As a global sequence modeling framework, this mechanism breaks through the local receptive field limitation of traditional temporal models by constructing a dynamic correlation weight matrix, enabling the modeling of nonlinear feature interactions between arbitrary interval time steps. Its core advantage lies in maintaining the sequence position awareness capability while capturing the dual temporal patterns of long-range periodicity and sudden volatility of traffic flow through a multihead parallel computing strategy.

For all nodes on the studied road section, the input to the temporal self-attention layer is a time feature sequence, as shown in Equation (9). This paper adopts the scaled dot-product attention mechanism in the Transformer architecture to achieve feature interaction and fusion across time steps. Meanwhile, to enhance the model’s ability to model temporal patterns, the temporal self-attention layer is configured with multiple groups of independent attention heads for parallel computation, as shown in Equation (10).

(9) $$Attention\left(Q,K,V\right)=Softmax\left(\frac{QK^{T}}{\sqrt{d_{k}}}+M_{mask}\right)V$$

In the equation, Q (Query), K (Key) and V (Value) are all derived from the same source input; $d_{k}$ is the dimension of the K vector.

(10) $$H^{\prime }=\left[\overset{n_{h}}{\underset{i=1}{\|}}Attention\left(HW_{q}^{\left(i\right)},HW_{k}^{\left(i\right)},HW_{v}^{\left(i\right)}\right)\right]W_{0}$$

In the equation, $n_{h}$ is the number of attention heads; $W_{q}^{\left(i\right)}$, $W_{k}^{\left(i\right)}$, $W_{v}^{\left(i\right)}$ are the learnable parameters of the i-th attention head; ‖ represents the concatenation operation.

Subsequently, tensor concatenation of multihead outputs is performed along the feature channel dimension, followed by a parameter matrix $W_{0}$ to finally achieve fusion and mapping to the original feature dimension.

#### 2.3.2. Design of Temporal Encoding

Temporal encoding serves as the positional embedding method for the temporal dimension of traffic flow data, embedding timestamp features independently of spatial topology. This encoding system possesses cross-node sharing properties, meaning that all nodes within the same time slice inherit identical temporal position identifiers. Temporal encoding consists of two components: absolute temporal encoding and relative temporal encoding, and its expression is shown in Equation (11).

(11) $$TEC=T{EC}_{a}+T{EC}_{r}$$

In the equation, $TEC\in R^{T\times h_{d}}$ is the temporal encoding, T is the number of time slices,${\;h}_{d}$ is the dimension of hidden layer features; $T{EC}_{a}$ is the absolute temporal encoding; $T{EC}_{r}$ is the relative temporal encoding.

This paper designs a corresponding absolute temporal encoding system for the periodic dependency characteristics related to absolute time in traffic flow, such as morning-evening peak fluctuations and weekly cycle pattern differences. In the feature engineering stage, the absolute time information of each time slice (such as intra-day period ordinal numbers, weekly ordinal numbers, etc.) is first extracted. Heterogeneous time dimensions are unified into the [0,1] standard dimension space through range normalization preprocessing to construct an absolute time feature tensor ${TF}_{a}\in R^{T\times S}$ with the dimension of S. Then, the absolute time features are transformed by a Multi-Layer Perceptron (MLP) to finally obtain the absolute temporal encoding $T{EC}_{a}$, as shown in Equation (12).

(12) $$T{EC}_{a}=MLP({TF}_{a},\theta_{T})$$

In the equation, ${TF}_{a}$ represents the absolute temporal features; $\theta_{T}$ is a learnable parameter.

The relative temporal encoding in this paper employs the sine–cosine encoding method proposed in Transformer, as shown in Equation (13).

(13) $$T{EC}_{r}\left(t,i\right)=\left\{\begin{array}{c} \sin\left(t/10000^{i/h_{d}}\right),\;\;\;\;\;\;\;\;\;\;i\;\mathrm{is}\;\mathrm{even}\\ \cos\left(t/10000^{(i-1)/h_{d}}\right),\;\;\;i\;\mathrm{is}\;\mathrm{odd} \end{array}\right.$$

This encoding is used to identify the relative position of a time slice within a sample or a time window (comprising T time slices), thereby informing the attention mechanism of the temporal order of the traffic flow sequence. By utilizing sine and cosine functions with different frequencies and phases to generate unique and regular encodings for different positions, the model can effectively capture the dependencies in the time series, thus enhancing its ability to model the temporal patterns of traffic flow. Algorithm 2 summarizes the temporal dependency modeling and parallel multistep prediction procedure.
**Algorithm 2.** Temporal dependency modeling and parallel multistep prediction**Input:** Fused feature sequence $\{z_{t}{\}}_{t=1}^{T_{in}}$, absolute temporal feature tensor ${TF}_{a}$, historical sequence length $T_{in}$, prediction horizon $T_{out}$, number of encoder layers $L_{e}$, and number of attention heads $n_{h}$.**Output:** Prediction tensor $\hat{Y}\in R^{T_{out}\times N\times 1}$.Map the fused feature sequence $\left\{z_{t}\right\}$ to the hidden dimension $h_{d}$ through a fully connected layer.Concatenate the projected historical features with the zero-padding matrix Z for the future $T_{out}$ time steps, where $T=T_{in}+T_{out}$.Transform ${TF}_{a}\;$ through the MLP to obtain the absolute temporal encoding ${TEC}_{a}\;$ according to Equation (12).Generate the relative temporal encoding ${TEC}_{r}$ using Equation (13).Combine the absolute and relative temporal encodings according to Equation (11).Add TEC to the concatenated feature representation to obtain the initial encoder input $H_{enc}^{\left(0\right)}$ according to Equation (4).Construct the temporal masking matrix $M_{mask}$ according to Equation (5), restricting attention to the current and previous time steps.**For** each encoder layer $l=0,\ldots ,L_{e}-1$ **do****For** each attention head $i=1,\ldots ,n_{h}$ **do**Calculate $Q_{i}=H_{enc}^{\left(l\right)}W_{q}^{\left(i\right)},K_{i}=H_{enc}^{\left(l\right)}W_{k}^{\left(i\right)},V_{i}=H_{enc}^{\left(l\right)}W_{v}^{\left(i\right)}.$Calculate the masked temporal attention: $O_{i}=\mathrm{Softmax}\left(\frac{Q_{i}K_{i}^{T}}{\sqrt{d_{k}}}+M_{mask}\right)V_{i}$**End for**Concatenate the outputs of all attention heads according to Equation (10).Apply the residual connection and layer normalization to $H^{\prime }$.Pass the normalized representation through the feedforward neural network, followed by another residual connection and layer normalization, to obtain $H_{enc}^{\left(l+1\right)}$.**End for**Map $H_{enc}^{\left(L_{e}\right)}$ to the dimension of all N road-network nodes through the output fully connected layer.Extract the representations corresponding to the future $T_{out}$ time steps to obtain $\hat{Y}$.Return $\hat{Y}$ as the parallel multistep traffic-flow prediction.

## 3. Data Processing and Experimental Design

### 3.1. Data Processing

#### 3.1.1. Data Source and Structure Description

The traffic flow data used in this study is derived from the actual operation data of G4202 Chengdu Ring Expressway. The road section is a bidirectional six-lane highway with a designed speed of 100 km/h. The data covers an 85 km long section, equipped with 46 gantries and 17 toll stations, collecting data from gantries and toll stations on the study section at 5 min intervals. The data collection period spans from 1 August 2024 to 8 November 2024, divided into three discontinuous time periods, with a cumulative record of 51 days. The daily data volume collected by gantries and toll stations on this section is approximately 18,100 records, including traffic flow, average speed, vehicle types, etc., and is highly representative and suitable for evaluating spatio-temporal prediction models.

In addition, based on the meteorological characteristics of high temperature, high humidity, and frequent precipitation in Chengdu during the study period, this paper selects weather and rainfall as the research objects to explore their potential impacts on traffic flow operation characteristics. The weather data used in this paper is collected from the China Meteorological Data Network, which provides national meteorological data with various types. By determining a point inside G4202 Ring Expressway through coordinates (104.1322, 30.7557), a total of 8760 pieces of weather and rainfall data at this point in 2024 are collected as the original weather dataset for this analysis.

#### 3.1.2. Data Preprocessing and Node Coverage Setting

The original traffic flow data contains a small number of missing values and outliers, which are corrected by the sliding window averaging method. To evaluate the traffic flow prediction capability of the model under different perception granularities, this paper designs various key node coverage settings and selects subset nodes based on the key ranking results to construct model inputs. The node ranking is based on the multifeature gravity model constructed in the early stage. This model integrates traffic flow characteristics and network topology factors, measures the influence of nodes on other nodes through gravitational values, and, thus, realizes the importance ranking of nodes. This method comprehensively considers traffic parameters such as traffic flow, average speed, density, and the “directivity” of road sections, and evaluates node importance by gravitational intensity. Since the focus of this paper is on the model structure and prediction effect, the node identification method is no longer described in detail, and the specific calculation logic can be referred to in relevant previous studies. Specifically, the node coverage rates are set to seven levels: 48%, 57%, 65%, 74%, 83%, 91%, and 100%, corresponding to different numbers of representative nodes.

#### 3.1.3. Spatio-Temporal-Weather Input Construction Under Sparse-Sensing Scenarios

After the key node set $V_{c}$ is determined, the traffic-flow sequences of the selected nodes, road-network topology, weather variables, and temporal-position information are organized as the input representation of STGFormer. This construction is consistent with the model architecture: the GCN module captures spatial dependencies among gantries, weather features describe external environmental disturbances, and temporal encoding together with self-attention captures temporal dependencies in traffic-flow sequences.

For the key node set $V_{c}$ under a given coverage rate c, a directed traffic graph is constructed as follows:

(14) $$G_{c}=(V_{c},E_{c},A_{c})$$

where $E_{c}$ denotes the directed edge set among key nodes, and $A_{c}$ denotes the corresponding adjacency matrix. The connections between gantry nodes are determined according to their spatial positions and traffic directions. Since highway traffic has clear upstream–downstream propagation characteristics, and traffic flows in opposite directions are physically separated by the median barrier, the A- and B-directions are modeled with separate spatial topologies. For gantry nodes connected in the same direction, the Gaussian kernel in Equation (6) is used to construct edge weights, so that closer gantries are assigned stronger spatial correlations, whereas distant or topologically unconnected gantries have weaker influence. Based on this graph structure, graph convolution is performed according to Equations (7) and (8). The two-layer GCN aggregates direct neighborhood information and higher-order neighborhood information, thereby preserving spatial dependency structures under sparse-sensing conditions.

Weather conditions and rainfall are incorporated as external disturbance variables. The raw weather data have a temporal resolution of 1 h, whereas the gantry traffic data are collected at 5 min intervals. Therefore, the weather records are aligned with the traffic-flow time slices to form weather features synchronized with the traffic-flow sequence. Discrete weather conditions are transformed into categorical vectors using one-hot encoding, while continuous variables such as rainfall are normalized. Let $q_{t}$ denote the weather category at time t, and let ${\tilde{r}}_{t}$ denote the normalized rainfall value. The weather feature vector is expressed as follows:

(15) $$w_{t}=[e(q_{t}),{\tilde{r}}_{t}]$$

where $e(q_{t})$ is the one-hot encoded vector corresponding to weather category $q_{t}$. Since the weather condition at the same time slice affects the entire study corridor, the weather feature vector is expanded along the node dimension and concatenated with the node-level spatial features extracted by the GCN along the feature dimension. This fusion process follows the feature concatenation operation in Equation (3), and the concatenated representation is further mapped by a fully connected layer to match the hidden dimension required by the Transformer encoder.

Timestamp information is used to supplement the temporal-position features of traffic-flow sequences. The raw timestamp field is not directly fed into the model as a string. Instead, it is decomposed into absolute temporal attributes, such as the intra-day time-slice index and the day-of-week index, to represent regular traffic patterns including morning and evening peaks, daily periodicity, and weekly periodicity. Meanwhile, the relative position of each time slice within the historical input window is represented using sinusoidal positional encoding. The temporal encoding consists of absolute temporal encoding and relative temporal encoding, as defined in Equations (11)–(13). Finally, the temporal encoding is added to the hidden traffic-flow representation according to Equation (4) before being fed into the self-attention module. This enables the model to distinguish traffic states with similar flow values but different temporal meanings.

In summary, the STGFormer input under coverage rate c consists of four types of information: the historical traffic-flow sequence of key nodes $X_{t-T_{in}+1:t}^{\left(c\right)}$, the key node traffic graph $G_{c}$, the weather-feature sequence aligned with the traffic-flow time slices, and the temporal-encoding sequence generated from timestamps. With this input construction, the model can jointly utilize road-network structure, weather disturbances, and temporal-position information while relying only on partial key node observations, thereby providing a unified spatio-temporal representation for traffic-flow prediction over all nodes.

### 3.2. Experimental Setup

#### 3.2.1. Experimental Environment and Parameter Settings

All experiments were completed under the Windows system, and the specific experimental environment is shown in Table 3.

To evaluate the experimental performance, this paper divides the dataset into training set, validation set and test set in chronological order, with the segmentation ratio of 7:1:2. The Adam optimizer and MAE loss are used for training, the batch size is set to 32, the initial learning rate is 0.001, and a total of 200 rounds of training are conducted. To prevent overfitting in training, the experiment adopts the strategy of early stopping, and the early stopping value is set to 10. To prevent model overfitting, the dropout ratio is set to 0.1. The hyperparameters are determined through grid search and validated on the validation set. The batch size of 32 balances training efficiency and gradient stability given the dataset size (51 days × 288 time steps). The initial learning rate of 0.001 is a standard choice for the Adam optimizer and prevents overshooting in early training. The maximum of 200 epochs with early stopping (patience = 10) ensures sufficient convergence while avoiding overfitting; in practice, training typically converges within 80–120 epochs. The dropout ratio of 0.1 provides mild regularization without degrading the model’s expressive capacity. The 12-step input window (60 min of history) captures short-term traffic dynamics while keeping computational cost manageable; preliminary experiments with 6-step and 24-step windows showed that 12 steps achieve the best trade-off between context length and prediction accuracy. Taking 5 min as a time step, this paper uses the data of the past 12 time steps as input to predict the data of future time steps. In addition, before the experiment, the input data is processed by the extreme value normalization method to process the data into the [0, 1] standard dimension space, so as to improve the numerical stability of the training process. The calculation process of the extreme value normalization method is shown in Equation (16).

(16) $$x_{i}^{\prime }=\frac{x_{i}-\min\left(x_{i}\right)}{\max\left(x_{i}\right)-\min\left(x_{i}\right)}$$

In Equation (16), $x_{i}^{\prime }$ is the normalized data; $x_{i}$ is the original data; $\max\left(x_{i}\right)$ is the maximum value of the original data; $\min\left(x_{i}\right)$ is the minimum value of the original data.

#### 3.2.2. Selection of Prediction Model Comparison Benchmark Models

To horizontally compare the prediction accuracy of the STGFormer model, this paper selects several classic prediction models as comparative experiments. These models can be divided into three categories: traditional statistical-based time series prediction models, deep-learning-based time series prediction models, and deep learning networks for traffic flow prediction by mining spatio-temporal features.

ARIMA (auto regression integrated moving average): The autoregressive integrated moving average model is relatively simple and essentially cannot capture the nonlinear relationship of data.

LSTM (long short-term memory): The long short-term memory network is a classic time series prediction network, which flattens traffic flow and uses it as input.

STGCN (spatial–temporal graph convolutional network): The spatio-temporal graph convolutional network consists of two spatio-temporal convolution blocks and an output layer. It uses graph convolution operations to capture spatial dependency and time convolution operations to mine dynamic changes in time series.

Transformer: By using the self-attention mechanism, it captures the global dependency of each element in the sequence. Unlike recurrent neural networks that process elements sequentially, it has no restrictions on the kernel size of convolutional neural networks. This model can capture both temporal and spatial dependencies, perform parallel computing, and improve efficiency.

To verify the prediction performance of the model, this paper selects three common indicators for evaluating models in deep learning: mean absolute error (MAE), mean absolute percentage error (MAPE), and root mean square error (RMSE).

### 3.3. Effectiveness Verification

**Figure 3:**
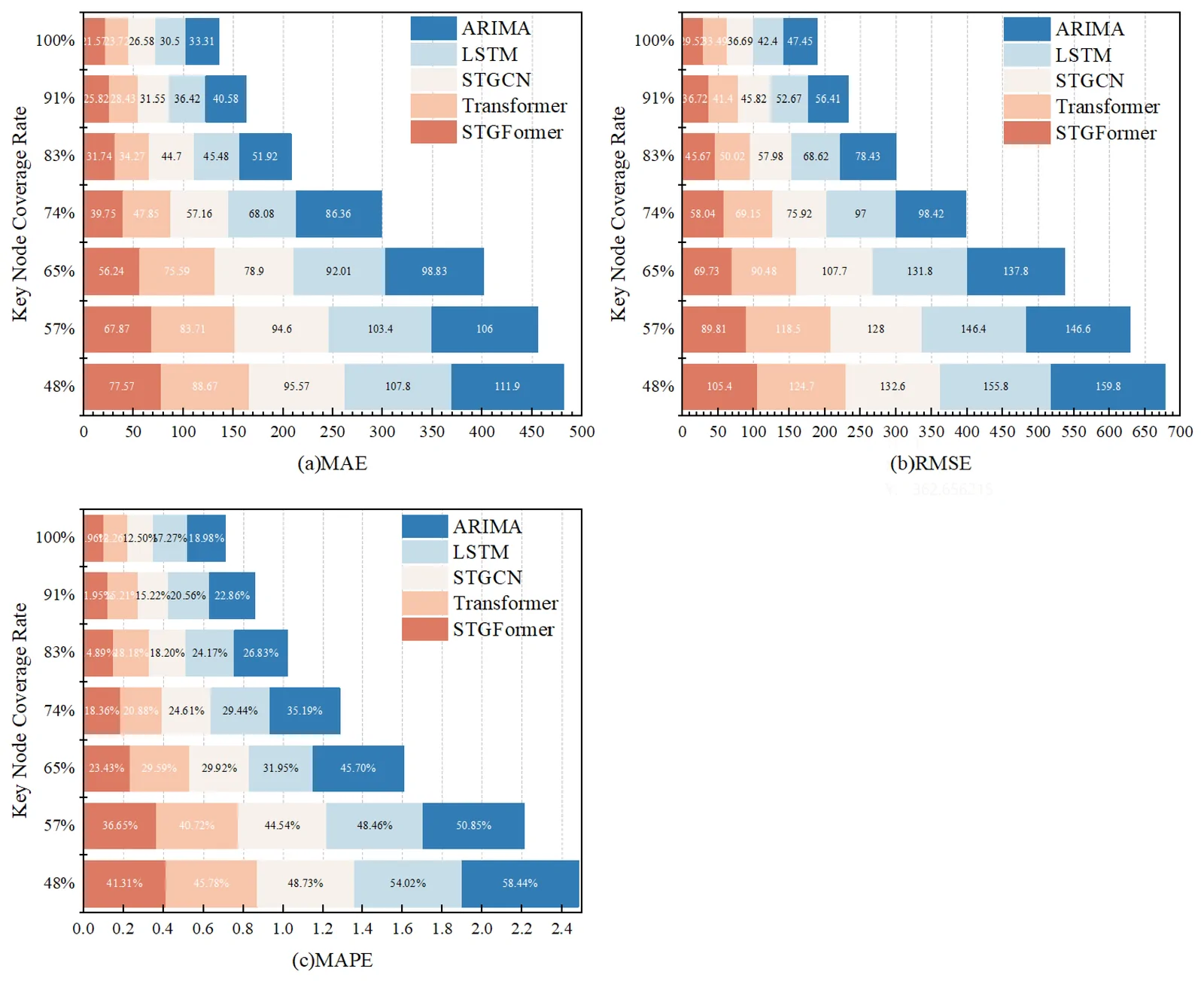
Performance comparison of each prediction model under different key node coverage rates.

The MAE, MAPE, and RMSE values of each prediction model under different node coverage rates are shown in Figure 3.

As shown in the figure, with the increase in key node coverage, the indicators of MAE, MAPE, and RMSE for all prediction models show a downward trend, indicating that the prediction performance of each model improves with the increase in key node coverage. This further verifies the universality of the proposed method, which uses traffic flow information from partial key nodes to predict the traffic flow information of all nodes in the study section.

From the perspective of the relative magnitudes of indicators for each prediction model under different node coverage rates, although the prediction performance of some models fluctuates with changes in key node coverage, the STGFormer model proposed in this paper consistently maintains the best prediction effect in terms of overall trend, followed by Transformer, STGCN, LSTM, and ARIMA.

Compared with the Transformer model, the STGFormer model improves prediction accuracy by an average of 3.72%, demonstrating the effectiveness of the improvements made to the Transformer model in this paper. In addition, due to the use of GCN and GLU to extract spatial and temporal features, STGCN also exhibits relatively good performance under different node coverage rates. Although the LSTM model achieves a certain level of prediction accuracy, it is more suitable for analyzing time series data, so its prediction effect for traffic data with spatial characteristics is not as excellent as the former. The traditional ARIMA model is more suitable for processing linear data and is not sensitive to the complex spatio-temporal structure data collected by highway detectors, ultimately resulting in the worst prediction effect.

When the key node coverage rate increases from 48% to 100%, the performance changes in each model are shown in Table 4. It can be seen from the table that among all prediction models, the STGFormer model has the smallest variation values in MAE, MAPE, and RMSE, indicating that changes in key node coverage have relatively little impact on the performance of the STGFormer model, which has strong stability.

### 3.4. Ablation Experiments

To further investigate the impacts of graph convolution, weather, and temporal features on the accuracy of the proposed traffic flow prediction model, this section sets up ablation experiments for the STGFormer model. We construct three derivative models by removing one component at a time:

1. STGFormer-weather: the full model without the weather-feature fusion module (Section 2.1, Equation (3));
2. STGFormer-GCN: the full model without the spatial feature extraction module (i.e., the dual-layer graph convolutional network in Section 2.2 is replaced by a simple linear projection);
3. STGFormer-TEC: the full model without the temporal encoding module (i.e., the learnable time encoding in Section 2.3.2, Equations (11)–(13), is removed).

The traffic flow prediction results of the above three derivative models and the full STGFormer under different node coverage rates are shown in Table 5.

**Figure 4:**
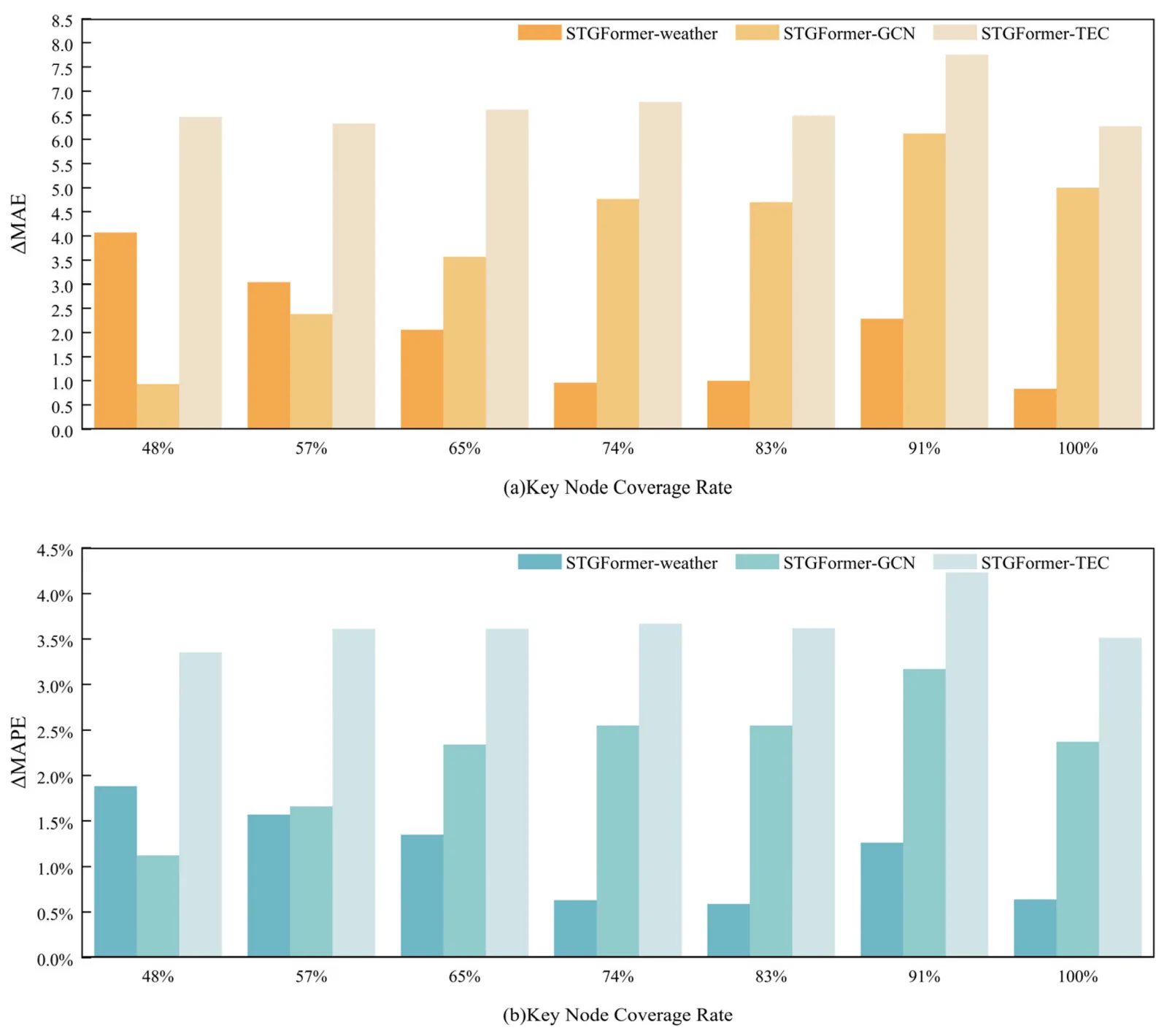
Differences in MAE and MAPE between derivative models and STGFormer model.

As shown in the data of Table 5, under different node coverage rates, the STGFormer model demonstrates the best prediction performance compared to the other three derivative models. To further analyze the contribution degree of each module, taking the MAE and MAPE indicators of the STGFormer model as the benchmark, the differences between the other three derivative models and the STGFormer model were calculated. The results are shown in Figure 4. The value of the vertical axis represents the contribution degree of the missing module in the corresponding derivative model under the corresponding node coverage rate; a larger value indicates a greater contribution.

The ablation study qualitatively demonstrates the cooperative mechanism among STGFormer’s components and quantitatively isolates each module’s contribution to prediction accuracy. Based on Figure 4, the following conclusions can be drawn:

1. From an overall perspective, the difference indicators of STGFormer-TEC are general-ly the largest, followed by STGFormer-GCN, while STGFormer-weather generally shows smaller difference indicators across the examined coverage rates. This indi-cates that the temporal encoding module contributes the most to the STGFormer model, followed by spatial features, and the weather factor module contributes the least.
2. Across the examined coverage rates, the MAE and MAPE differences between STGFormer-TEC and the STGFormer model remain generally high despite some fluctuations, indicating that the temporal encoding module maintains a relatively strong contribution. Analyzing the characteristics of this feature, since key nodes mainly contain spatial features, which have little impact on temporal encoding, the temporal encoding module generally maintains a high contribution as the key node coverage rate increases. Additionally, the introduction of temporal encoding enhances the model’s ability to capture temporal correlations, working in conjunction with existing spatial features to enable the model to capture spatio-temporal characteristics, thereby significantly improving prediction accuracy.
3. With the increase in key node coverage rate, the MAE and MAPE differences between STGFormer-GCN and the STGFormer model show an overall increasing tendency with some fluctuations, indicating that spatial features become increasingly im-portant at medium-to-high coverage rates. From this trend, it can be inferred that when the key node coverage rate is low, the limited number of key nodes makes it more difficult to capture road network spatial features, resulting in limited contribution of spatial features to the model. As the key node coverage rate increases, spatial features can more easily identify the spatial characteristics between nodes, and their contribution begins to rise. When the number of key nodes is sufficient, these key nodes can capture more comprehensive spatial features in the road network, and the contribution of spatial features remains relatively high.
4. As the key node coverage rate increases, the MAE and MAPE differences between STGFormer-weather and the STGFormer model show an overall decreasing tendency with local fluctuations, indicating that the relative contribution of weather factors is generally more evident under low-coverage conditions. Analyzing the characteristics of this feature, when the key node coverage rate is low, the impact of weather factors on local nodes is amplified, making their contribution to the model relatively large. As the key node coverage rate increases, the impact of weather factors is continuously diluted by the increasing number of key nodes, leading to a decreasing contribution. When the impact of weather factors is diluted to a certain extent, their contribution remains relatively limited at higher coverage rates.

The above ablation experiments explore the contribution of each module to the model. Modeling temporal and spatial dependencies can effectively improve the model’s prediction accuracy, where spatial features further enhance prediction accuracy under high key node coverage, and weather factors compensate for prediction accuracy under low key node coverage.

In summary, the quantitative margins exposed in the ablation trials exhibit a profound convergence with the input tensor construction framework detailed in Section 3.1.3. The temporal encoding module yields the most substantial contribution to error reduction because it reconstructs the core historical periodic invariants for the 5 min sequences. The GCN-based spatial convolutions manifest escalating importance as the key node coverage rate scales up, enabling the network to effectively aggregate multihop topological features. Meanwhile, the meteorological integration compensates for information degradation specifically within low-coverage regions by infusing stable environmental constraints. The perfect alignment between the input block engineering and the mathematical ablation outcomes firmly verifies the structural logic of the proposed STGFormer paradigm.

## 4. Conclusions and Discussion

This study addresses the inherent limitations of Transformer-based models in spatial modeling and their lack of environmental adaptability for traffic flow prediction by proposing STGFormer, an improved framework integrating graph convolutional networks (GCNs) with a self-attention mechanism. By incorporating cascaded GCN modules to extract non-Euclidean spatial features and learnable time encoding to capture long-term and abrupt temporal fluctuations, the model establishes a unified framework for spatio-temporal dynamic awareness. To address the practical constraint of uneven sensing resource distribution, a key node coverage paradigm was evaluated. Quantitatively, STGFormer effectively infers global traffic states from sparse local observations. It achieves an MAE/MAPE/RMSE of 21.57/9.96%/29.52 at 100% node coverage and maintains robust performance (77.57/41.31%/105.39) even at 48% sparse input. The model outperforms the Transformer baseline by an average of 3.72% in prediction accuracy and exhibits superior stability across varying coverage rates (ΔMAPE = 31.34%) compared to STGCN (36.23%), LSTM (36.75%), and ARIMA (39.46%). Furthermore, by innovatively integrating meteorological variables into the prediction process from heterogeneous data sources, the model achieves high adaptability in complex environments. Ablation studies elucidate the cooperative mechanism among the model’s components: temporal encoding plays the most pivotal role in capturing periodic traffic patterns; spatial feature extraction proves highly effective under medium- to high-coverage settings; and weather factors critically compensate for local information loss in low-coverage scenarios.

Despite these advancements, accurately capturing and fully exploiting topological relationships in the monitored traffic network remains a complex challenge, presenting certain limitations in this study. First, the model relies on a predefined road topology derived from inter-gantry distances, which may not fully capture dynamic spatial dependencies such as time-varying congestion propagation. Second, applying multihop message passing in extremely sparse scenarios (coverage < 40%) remains an open challenge, as the limited number of observed nodes restricts the structural information available for reliable inference. Third, the current integration of coarse hourly weather data limits the model’s sensitivity to sudden micro-meteorological changes. Future work will focus on deepening the fusion of multisource heterogeneous data, including dynamic coupling of refined meteorological parameters (e.g., visibility, road surface conditions) and real-time traffic events (e.g., accidents, road closures), to improve robustness under extreme scenarios. Additionally, the generalization of the model will be tested on more complex topologies such as large-scale interchanges and urban road networks, addressing the challenge of modeling high-dimensional non-Euclidean spatial relations. Finally, by integrating with the vehicle-to-everything (V2X) ecosystem, future research will explore a novel predictive paradigm driven by connected vehicle trajectories and roadside sensing data, advancing intelligent transportation systems toward a fully integrated perception-decision framework.

## Figures and Tables

**Table 1:** Comparison of representative traffic prediction studies.

Reference	Model	Core Modeling Feature	Sparse Sensing and External Factors
Li et al. [4]	Conv–BiLSTM	CNN-based spatial extraction and bidirectional temporal modeling	Explicit graph topology and sparse key-node coverage are not specifically addressed
Yang et al. [5]	Multi-feature CNN	Fusion of temporal continuity, daily/weekly periodicities, weather, and holiday features	Explicit graph topology and sparse key-node coverage are not evaluated
Zhao et al. [9]	T-GCN	GCN-based spatial modeling and GRU-based temporal modeling	Sparse key-node coverage is not evaluated
Zheng et al. [11]	GMAN	Spatiotemporal attention and transform attention	Weather variables and sparse key-node coverage are not specifically addressed
Yu et al. [13]	STGCN	Graph convolution and temporal convolution	Weather-feature fusion and sparse sensing are not jointly considered
Li et al. [19]	DDGformer	Direction- and distance-aware graph Transformer	Weather-feature fusion and sparse key-node coverage are not the reported focus
This study	STGFormer	GCN, temporal self-attention, and temporal encoding	Weather-aware full-network prediction across seven key-node coverage rates; best overall performance among the implemented baselines

**Table 2:** Nomenclature of principal symbols and indices.

Symbol	Description
*X*	Historical traffic-flow input tensor
$\hat{Y}$	Predicted traffic-flow tensor
T	Total time-window length
$T_{in},T_{out}$	Numbers of input and prediction time steps
$N,N_{k}$	Numbers of all nodes and selected key nodes
F	Number of input features
${c,V}_{c}$	Key-node coverage rate and selected key-node set
$G_{c}=(V_{c},E_{c},A_{c})$	Traffic graph constructed from the selected key nodes
$A_{c},\tilde{A_{c}},\tilde{D_{c}}$	Weighted adjacency, self-connected adjacency, and degree matrices
L	Renormalized graph matrix used in graph convolution
$H_{gcn,t}^{\left(l\right)}$	Node representation at the $l^{th}$ GCN layer and time step t
$H_{enc}^{\left(l\right)}$	Hidden representation at the $l^{th}$ Transformer encoder layer
$h_{d}$	Hidden-feature dimension
$S^{W},w_{t}$	Weather-feature sequence and weather vector at time step t
$q_{t},{\tilde{r}}_{t}$	Weather category and normalized rainfall at time step t
$z_{t}$	Fused spatial–weather feature at time step t
$TEC,{TEC}_{a},{TEC}_{r}$	Overall, absolute, and relative temporal encodings
Q,K,V	Query, key, and value matrices
$M_{mask}$	Temporal masking matrix
Z	Zero-padding matrix
$n_{h},d_{k}$	Number of attention heads and dimension of query/key vectors
$L_{e}$	Number of Transformer encoder layers

**Table 3:** Experimental environment.

Experimental Environment	Specification
Operating System	Windows 11, 64-bit
CPU	AMD Ryzen 7 8845H
GPU	NVIDIA GeForce RTX 4070 Laptop GPU (NVIDIA Corporation, Santa Clara, CA, USA)
Deep Learning	Pytorch-2.5.1+cu124
CUDA	12.7

**Table 4:** Variation values of MAE, MAPE and RMSE for different prediction models.

Index	Coverage Rate	STGFormer	Transformer	STGCN	LSTM	ARIMA
MAE	48%	77.57	88.67	95.57	107.80	111.92
	100%	21.57	23.72	26.58	30.50	33.31
	Difference Value	56.00	64.95	69.00	77.29	78.61
MAPE	48%	41.31%	45.78%	48.73%	54.02%	58.44%
	100%	9.96%	12.26%	12.50%	17.27%	18.98%
	Difference Value	31.34%	33.52%	36.23%	36.75%	39.46%
RMSE	48%	105.39	124.66	132.61	155.82	159.83
	100%	29.52	33.49	36.69	42.40	47.45
	Difference Value	75.87	91.17	95.91	113.42	112.38

**Table 5:** Comparison table of prediction performance for STGFormer model ablation experiments.

Coverage Rate	STGFormer		STGFormer-Weather		STGFormer-GCN		STGFormer-TEC	
	MAE	MAPE	MAE	MAPE	MAE	MAPE	MAE	MAPE
48%	77.57	41.31%	81.64	43.19%	78.50	42.43%	84.04	44.66%
57%	67.87	36.65%	70.91	38.22%	70.25	38.31%	74.20	40.26%
65%	56.24	23.43%	58.30	24.78%	59.81	25.77%	62.85	27.04%
74%	39.75	18.36%	40.71	18.99%	44.52	20.91%	46.52	22.03%
83%	31.74	14.89%	32.74	15.48%	36.44	17.44%	38.23	18.51%
91%	25.82	11.95%	28.11	13.21%	31.94	15.12%	33.58	16.18%
100%	21.57	9.96%	22.41	10.60%	26.57	12.33%	27.84	13.47%

## Data Availability

The authors do not have permission to share.

## Abbreviations

The following abbreviations are used in this manuscript:

GCN	Graph Convolutional Network
ITS	Intelligent Transportation Systems
STPGNN	Spatio-Temporal Pivotal Graph Neural Network
SVR	Support Vector Regression
RNNs	Recurrent Neural Networks
CNNs	Convolutional Neural Networks
BiLSTMs	Bidirectional Long Short-Term Memory networks
GCNs	Graph Convolutional Networks
DSTHGCN	Dynamic Spatio-temporal Hypergraph Convolutional Network
STGFormer	Spatio-Temporal Graph Transformer
FFN	Feedforward Neural Network
ARIMA	Autoregressive Integrated Moving Average
LSTM	Long Short Term Memory
STGCN	Spatial–Temporal Graph Convolutional Network
